# Changes in SARS-CoV-2 Spike versus Nucleoprotein Antibody Responses Impact the Estimates of Infections in Population-Based Seroprevalence Studies

**DOI:** 10.1128/JVI.01828-20

**Published:** 2021-01-13

**Authors:** Craig Fenwick, Antony Croxatto, Alix T. Coste, Florence Pojer, Cyril André, Céline Pellaton, Alex Farina, Jérémy Campos, David Hacker, Kelvin Lau, Berend-Jan Bosch, Semira Gonseth Nussle, Murielle Bochud, Valerie D’Acremont, Didier Trono, Gilbert Greub, Giuseppe Pantaleo

**Affiliations:** a Service of Immunology and Allergy, Department of Medicine, Lausanne University Hospital and University of Lausanne, Lausanne, Switzerland; b Institute of Microbiology, Lausanne University Hospital and University of Lausanne, Lausanne, Switzerland; c Service of Infectious Diseases, Lausanne University Hospital, Lausanne, Switzerland; d Virology Section, Infectious Diseases and Immunology Division, Department of Biomolecular Health Sciences, Faculty of Veterinary Medicine, Utrecht University, Utrecht, The Netherlands; e Protein Production and Structure Core Facility, School of Life Sciences, École Polytechnique Fédérale de Lausanne (EPFL), Lausanne, Switzerland; f School of Life Sciences, Ecole Polytechnique Fédérale de Lausanne, Lausanne, Switzerland; g Centre for Primary Care and Public Health, University of Lausanne, Lausanne, Switzerland; h Swiss Vaccine Research Institute, Lausanne University Hospital and University of Lausanne, Lausanne, Switzerland; The Peter Doherty Institute for Infection and Immunity

**Keywords:** SARS-CoV-2, serology, S protein trimer

## Abstract

Severe acute respiratory syndrome coronavirus 2 (SARS-CoV-2)-specific antibody responses to the spike (S) protein monomer, S protein native trimeric form, or the nucleocapsid (N) proteins were evaluated in cohorts of individuals with acute infection (*n* = 93) and in individuals enrolled in a postinfection seroprevalence population study (*n* = 578) in Switzerland. Commercial assays specific for the S1 monomer, for the N protein, or within a newly developed Luminex assay using the S protein trimer were found to be equally sensitive in antibody detection in the acute-infection-phase samples. Interestingly, compared to anti-S antibody responses, those against the N protein appear to wane in the postinfection cohort. Seroprevalence in a “positive patient contacts” group (*n* = 177) was underestimated by N protein assays by 10.9 to 32.2%, while the “randomly selected” general population group (*n* = 311) was reduced by up to 45% relative to the S protein assays. The overall reduction in seroprevalence targeting only anti-N antibodies for the total cohort ranged from 9.4 to 31%. Of note, the use of the S protein in its native trimer form was significantly more sensitive compared to monomeric S proteins. These results indicate that the assessment of anti-S IgG antibody responses against the native trimeric S protein should be implemented to estimate SARS-CoV-2 infections in population-based seroprevalence studies.

**IMPORTANCE** In the present study, we have determined SARS-CoV-2-specific antibody responses in sera of acute and postinfection phase subjects. Our results indicate that antibody responses against viral S and N proteins were equally sensitive in the acute phase of infection, but that responses against N appear to wane in the postinfection phase where those against the S protein persist over time. The most sensitive serological assay in both acute and postinfection phases used the native S protein trimer as the binding antigen, which has significantly greater conformational epitopes for antibody binding compared to the S1 monomer protein used in other assays. We believe these results are extremely important in order to generate correct estimates of SARS-CoV-2 infections in the general population. Furthermore, the assessment of antibody responses against the trimeric S protein will be critical to evaluate the durability of the antibody response and for the characterization of a vaccine-induced antibody response.

## Abstract (precis)

In the present study, we have determined SARS-CoV-2-specific antibody responses in sera of acute and postinfection phase subjects. Our results indicate that antibody responses against viral S and N proteins were equally sensitive in the acute phase of infection, but that responses against N appear to wane in the postinfection phase where those against the S protein persist over time. The most sensitive serological assay in both acute and postinfection phases used the native S protein trimer as the binding antigen, which has significantly greater conformational epitopes for antibody binding compared to the S1 monomer protein used in other assays. We believe these results are extremely important in order to generate correct estimates of SARS-CoV-2 infections in the general population. Furthermore, the assessment of antibody responses against the trimeric S protein will be critical to evaluate the durability of the antibody response and for the characterization of a vaccine-induced antibody response.

## INTRODUCTION

The severe acute respiratory syndrome coronavirus 2 (SARS-CoV-2) is currently causing a devastating pandemic, with more than 12.7 million documented infections and more than 566,000 deaths, according to the latest WHO situation report from 13 July 2020 (1). However, the true incidence of the infection is largely underestimated, since in most countries asymptomatic and paucisymptomatic people are tested only if they come in direct contact with sick patients or belong to at-risk subgroups. Therefore, there is a public health urgency to perform large-scale population-based studies in order to determine rates of seroprevalence during the first wave of the SARS-CoV-2 pandemic and to implement continued surveillance with the combined use of viral detection tests such as reverse transcriptase PCR (RT-PCR) and serological testing. Seroprevalence studies are also instrumental to determine the proportion of individuals with potential protective immunity (2–4).

SARS-CoV-2 antibody responses are characterized through the detection of IgG, IgA, and/or IgM. Detecting both IgA and IgG may increase sensitivity, particularly for people experiencing paucisymptomatic or asymptomatic infection (5). In contrast, IgM does not seem to be of great benefit to overall sensitivity, since IgM appearance coincides with IgG antibodies during the early phase of infection, i.e., less than 15 days after the onset of symptoms, and may increase the likelihood of false-positive results due to cross-reactivity (6, 7).

SARS-CoV-2-specific antibody responses target two proteins: the nucleocapsid protein (N) and the spike protein (S). It has been suggested that IgG antibodies targeting the S protein are more specific, while those targeting N may be more sensitive, particularly in the early phase of infection (8).

However, the increased sensitivity of anti-N antibody response might be at the expense of specificity, given the relatively high protein sequence similarity of the N protein of SARS-CoV-2 with nucleocapsid proteins of other members of the *Coronaviridae* and other virus groups (8, 9). Moreover, during the first SARS outbreak (2002 to 2004), Chia et al. observed that anti-N antibodies waned earlier than anti-S antibodies (10). Thus, the anti-S antibody response might be more specific and circumvent a possible decrease of antibodies, as was previously observed with the N protein of the SARS virus. Further, the durability of the SARS-CoV-2 antibody response is still unknown. Previous studies have shown early disappearance of antibodies to SARS-associated coronavirus after recovery (11), while other studies have shown longer durability of the antibody response (12–15).

In the present study, we have investigated SARS-CoV-2 antibody responses, both IgA and IgG, in a cohort of 93 patients with moderate to severe symptoms during the first 33 days of the acute phase of infection, and in a cohort of 578 subjects mostly paucisymptomatic and/or asymptomatic enrolled in a population-based seroprevalence study of the Vaud Canton in Switzerland. Antibody responses targeting either the N and/or the S proteins were investigated. Anti-S antibody responses were determined against monomeric moieties of the S1 protein and/or the native S trimeric form. Antibody responses against the S and N proteins were equally sensitive during the acute phase of infection, while the anti-N antibody responses waned in the postinfection phase. Importantly, the use of the trimeric compared to the monomeric form of the S protein was associated with greater sensitivity in the detection of SARS-CoV-2 IgG antibody response in both the acute and postinfection phases.

Taken together, these results indicate that antibody responses against the native trimeric S protein should be used as a reference in population-based seroprevalence studies to provide more accurate estimates of SARS-CoV-2 infections in the general population.

## RESULTS

### Antibody responses in a Luminex immunoassay developed using native trimeric S protein.

**FIG 1:**
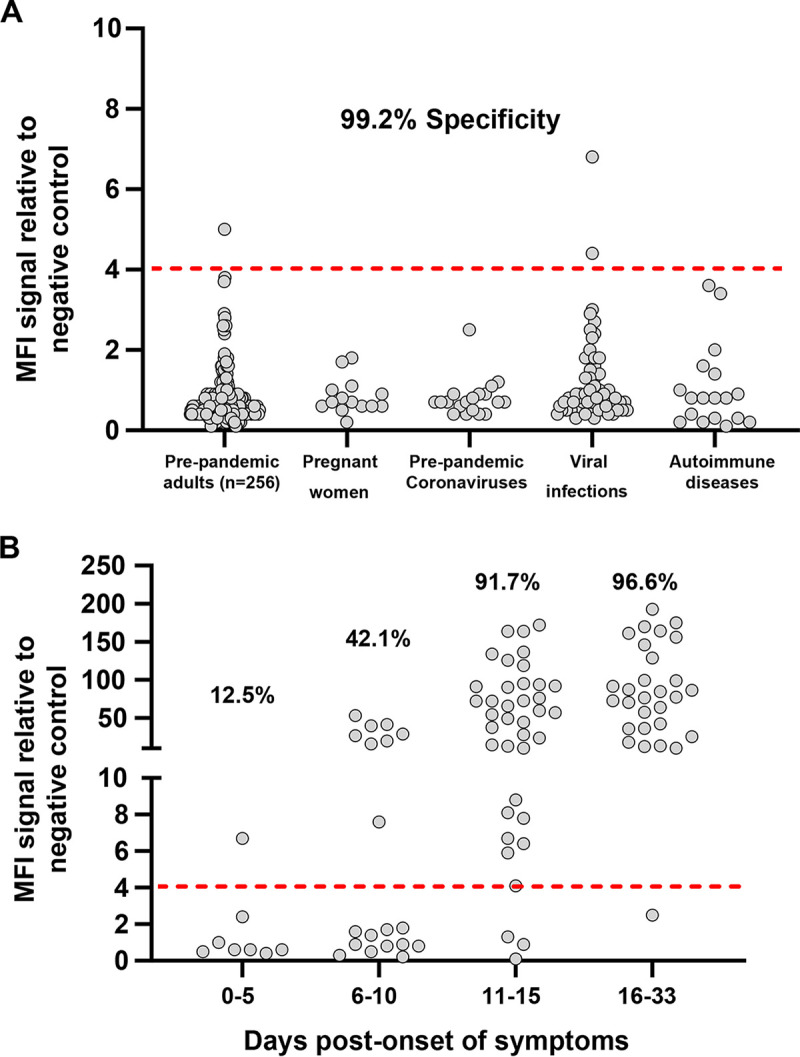
SARS-CoV-2-specific IgG binding antibody responses against the native trimeric S protein in a Luminex binding assay. Luminex beads covalently coupled with SARS-CoV-2 S protein trimer were used to monitor IgG-binding antibody responses in pre-COVID-19 pandemic negative-control sera and sera from SARS-CoV-2 PCR-positive donors. MFI signals for serum antibody binding were expressed as ratios compared to a negative-control pool of pre-COVID-19 pandemic human serum tested in parallel. (A) Assay specificity was evaluated using the sera from pre-COVID-19 pandemic healthy adults (*n* = 256; ages ranging between 18 to 81 years of age), pregnant woman (*n* = 14), prepandemic coronavirus-infected donors (OC43, E229, NL63, and HKU1; *n* = 19), patients with infectious diseases (HIV, rubella, HSV1, HSV2, RSV, CMV, EBV, influenza, and varicella; *n* = 57), and patients with autoimmune diseases, including lupus (*n* = 18). (B) The sensitivity of the S protein trimer was evaluated with sera from acute-infected SARS-CoV-2 PCR-positive donors at 0 to 5 days, 6 to 10 days, 11 to 15 days, and 16 to 33 days post-onset of symptoms. The red dashed line in A and B corresponds to the 4.0 cutoff for positivity in the IgG Luminex assay that was established by using mean value + (4 × SD) of all 364 pre-COVID-19 pandemic serum samples shown in A.

A stabilized trimer of the full-length S protein, encompassing both its S1 and S2 moieties, was coupled to beads for capturing antibodies in a new Luminex assay. We hypothesized that conformational epitopes would be preserved in the trimeric S protein, providing a greater sensitivity to detect IgG antibodies (Fig. S1A and B in the supplemental material) (16). First, the specificity for IgG antibody binding was established with sera from 256 pre-COVID-19 pandemic healthy adults from 18 to 81 years of age and an additional set of 108 patients (Fig. 1A) that included pregnant women, individuals infected with alphacoronaviruses (NL63 and 229E), betacoronaviruses (OC43 and HKU1), HIV, rubella virus, herpes simplex virus 1 (HSV1), HSV2, respiratory syncytial virus (RSV), cytomegalovirus (CMV), Epstein-Barr virus (EBV), influenza virus, or varicella-zoster virus, as well as patients suffering from autoimmune diseases, such as lupus. The signal distribution for all SARS-CoV-2-negative sera was similar for the 256 pre-COVID-19 healthy adults and for the diverse panel of 108 subjects. A cutoff for positivity was set at 4-fold above a negative-control standard, which is slightly more than four standard deviations (SD) above the mean of all negative-control samples (mean fluorescence intensity [MFI] ratio 0.84 + [4 × 0.75 SD]). Using this threshold, only one serum of the 256 pre-COVID-19 people and two patients with acute HIV or CMV viral infections gave a positive signal (Fig. 1A). As such, the Luminex assay using the stable trimeric S protein gave a high overall specificity of 99.2% and no cross-reactive antibodies were detected in sera from people infected with prepandemic coronaviruses or from patients with autoimmune diseases that can produce polyreactive antibodies.

The sensitivity of the assay was next evaluated using sera from 93 acutely infected SARS-CoV-2 PCR-positive patients with blood sampling at 0 to 5 days, 6 to 10 days, 11 to 15 days, and 16 to 33 days post-onset of symptoms (POS). As anticipated, sera collected during the early stage of the infection (0 to 5 days POS) had low or undetectable levels of anti-S protein IgG antibodies, with a rate of positivity of 12.5% (1 in 8 subjects; Fig. 1B). Seropositivity increased to 42.1% (8/19) at 6 to 10 days POS and to 91.7% (33/36) at 11 to 15 days POS. Almost all patients with symptoms for 16 to 33 days (28/29; 96.6%) displayed high antibody titers for the S protein trimer. Interestingly, the only subject who was negative in the S protein trimer assay at day 25 post-onset of symptoms became seropositive when retested 7 days later.

We then performed head-to-head comparisons of the trimeric S versus S1 or the receptor-binding domain (RBD) monomeric proteins for IgG antibody responses within the Luminex assay. The responses observed with the S monomeric proteins were similar in sensitivity to those described in previous studies (17, 18) using monomeric proteins, but inferior to those obtained with the trimeric S protein (Fig. S2A and B).

### Anti-IgA antibody response against the trimeric S protein.

**FIG 2:**
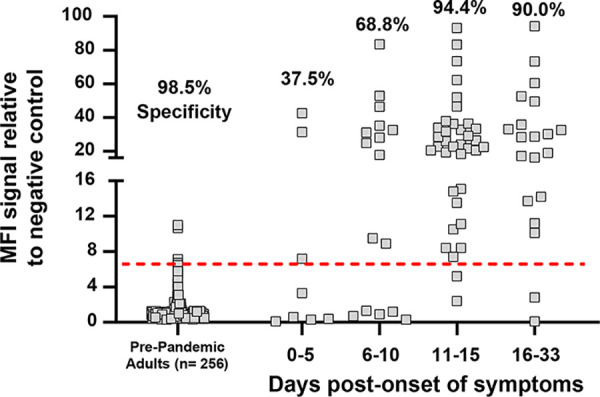
SARS-CoV-2-specific IgA-binding antibody responses against the native trimeric S protein in a Luminex binding assay. The native trimeric S protein was used to monitor IgA-binding antibodies in sera from pre-COVID-19 pandemic negative-control donors and sera from acute SARS-CoV-2 PCR-positive donors. The Luminex assay exhibited high specificity of 98.5% against a cohort of 256 prepandemic adults (left side of graph) and was effective at detecting IgA antibodies specific for S protein in most subjects at both the early stage (0 to 10 days) and later stage (11 to 33 days) post-onset of symptoms in acute PCR-positive patients (right side of graph). The red dashed line corresponds to a 6.5-fold MFI signal over the internal negative control and was established by using the mean value + (4 × SD) of the 256 pre-COVID-19 pandemic adult serum samples.

We next evaluated the S protein trimer for the detection of anti-SARS-CoV-2 IgA antibodies. We established assay specificity and a cutoff threshold for positivity by screening sera from pre-COVID-19 healthy adults. Using four standard deviations above the standard negative control, this assay provided a 98.5% specificity in the 256 sera tested. The sensitivity was estimated on 81 out of 93 acute-infected SARS-CoV-2 patients’ sera, with positive detections ranging from 37.5% of patients at 0 to 5 days POS and seropositivity increasing to 68.8% in patients from the 6 to 10 days group. At 11 to 15 and 16 to 33 days POS, IgAs were detected in 94.4% and 90% of the cases, respectively (Fig. 2).

### Comparison of Luminex anti-S trimer and commercial anti-S and anti-N protein assays in the detection of antibody responses during the acute phase of infection.

Anti-S and anti-N antibody responses were determined using four different technologies, i.e., Luminex, enzyme-linked immunosorbent assay (ELISA), chemiluminescent immunoassay (CLIA), and electro-chemiluminescence immunoassay (ECLIA). The different assays used were detecting the N protein alone, the N plus a monomeric antigen of the S protein, the monomeric S1 protein alone, and the native trimeric S protein. More details about the five commercial assays used are contained in the Materials and Methods. We performed the comparison on the same set of 93 sera from patients with acute infection and stratified based on time between symptom onset and sera collection, as shown in Fig. 1B. Small differences in the number of sera tested across assays were due to the insufficient volume of some samples. The specificity was evaluated on a common panel of 65 pre-COVID-19 pandemic sera sampled before November 2019.

**FIG 3:**
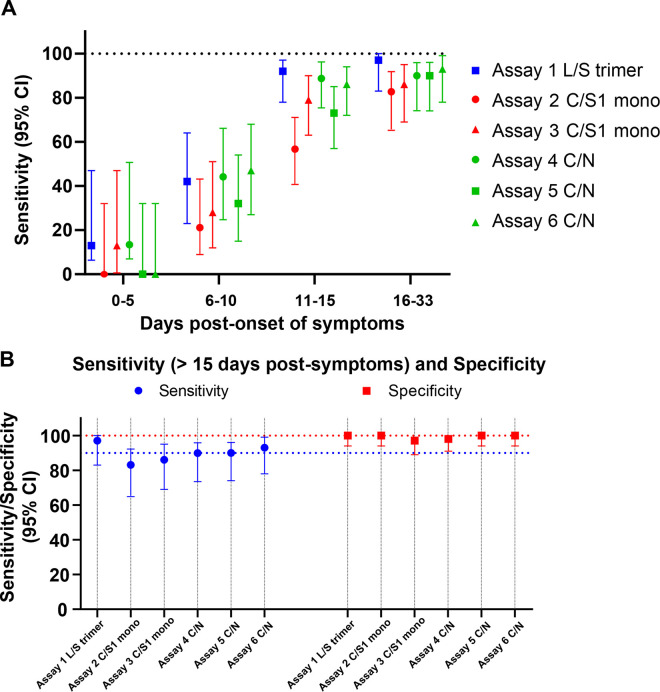
Comparative analysis of SARS-CoC-2-specific IgG binding antibody responses against S and N proteins in sera from patients with acute infection using six different serological assays. Sensitivity in detecting anti-SARS-CoV-2-specific IgG antibodies was assessed using the Luminex assay and five other commercial assays as described in the Materials and Methods section. (A) Serum samples were grouped by the number of days post initial onset of symptoms with sensitivity increasing over time. (B) Comparison in sensitivity between the different assays in samples collected from day 16 to 33 post-symptoms. L/S trimer, Luminex assay with S trimer antigen; C/S1 mono, commercial assay with S1 monomer antigen; C/N, commercial assay with nucleocapsid protein antigen.

Increased sensitivity in the detection of both anti-N and anti-S IgG antibody responses was observed consistently over time post-onset of symptoms regardless of the assay used (Fig. 3A, Table S1). The use of the native trimeric S protein (assay 1 L/S trimer) was associated with the higher sensitivity, i.e., detection of anti-S IgG antibodies in 97% of individuals tested at >15 days POS, compared to the use of monomeric S (assays 2 and 3 C/S1 mono) and/or N proteins (assays 4 to 6 C/N), with a sensitivity ranging between 83% to 93% (Fig. 3A, Table S1). The specificity was equal to or higher than 97% (Fig. 3B) regardless of the assay and antigen used and none displayed cross-reactivity with sera from patients positive for 229E, OC43, HKU1, or NL63 coronaviruses (Fig. 1A). Taken together, these results indicate that antibody responses targeting the S and/or the N proteins have similar sensitivity during the acute phase of infection.

### Comparison of antibody responses in the Luminex anti-S trimer assay with commercial anti-S monomer and anti-N IgG antibody responses during the postinfection phase.

**FIG 4:**
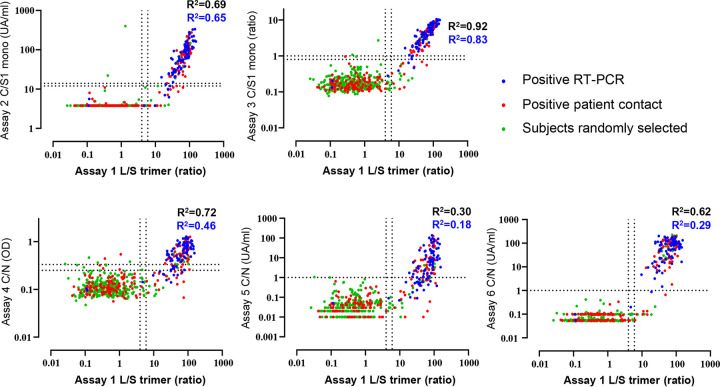
Comparative analysis of IgG antibody responses against the trimeric S protein versus monomeric S and/or N proteins. Signal intensities for the different subject sera in the postinfection cohort were compared between the Luminex assay and the five other serological assays. Collected sera were from patients with a documented positive SARS-CoV-2 RT-PCR (90 sera; blue dots), positive patient contacts with a SARS-CoV-2 RT-PCR positive patient (177 sera; red dots), and randomly selected subjects from the general population (311 sera; green dots). Pearson correlation *R*^2^ values are given for all 578 participants (black text) or for the 183 Luminex positive sera (blue text). Regions between the dotted lines in individual graphs are indeterminate limits of response in each of the assays and above the upper limit dotted line corresponds to seropositive samples.

Next, while blind to the seropositivity status, we evaluated anti-S and anti-N IgG antibody responses on 578 sera taken as part of a population-based seroprevalence study of the Vaud Canton in Switzerland. These comparisons included 90 sera sampled from mildly to paucisymptomatic patients tested positive by RT-PCR, 177 sera sampled from “positive patient contacts” of RT-PCR-positive subjects, and 311 sera sampled from undefined randomly selected people from the general population of ages 6 months and over. Results of the comparisons are shown in Fig. 4 and Table 1. As expected, a good correlation in the proportion of seropositive individuals was observed between assays detecting antibody responses against the trimeric and/or monomeric S proteins, while a poorer correlation was observed with those detecting anti-N antibody responses (Fig. 4).

**TABLE 1:**
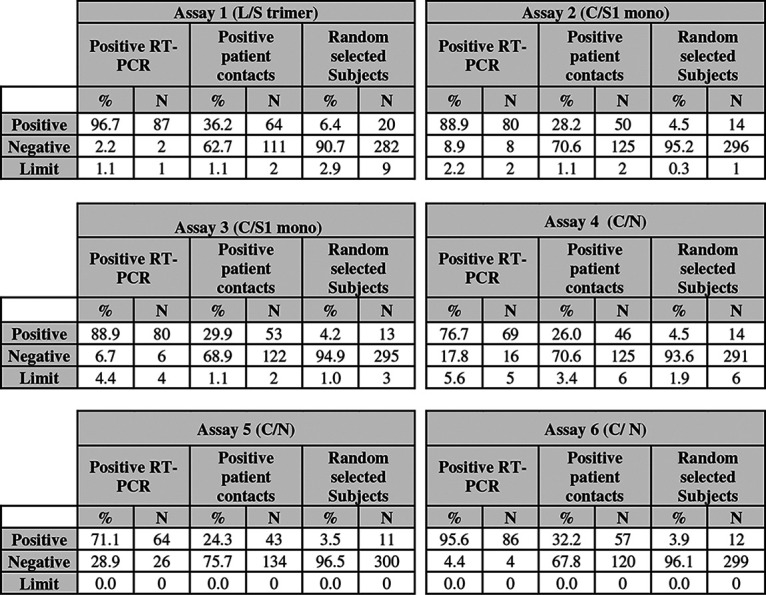
Estimates of SARS-CoV-2 seropositive subjects in the postinfection population-based study^*a*^

a Sensitivity on positive RT-PCR sera: Assay 1 (L/S trimer) 95% CI: 0.92 to 0.996; Assay 2 (C/S1 mono) 95% CI: 0.83 to 0.95; Assay 3 (C/S1 mono) 95% CI: 0.86 to 0.97; Assay 4 (C/N) 95% CI: 0.72 to 0.88; Assay 5 (C/N) 95% CI: 0.61 to 0.79; Assay 6 (C/N) 95% CI: 0.89 to 0.98.

**FIG 5:**
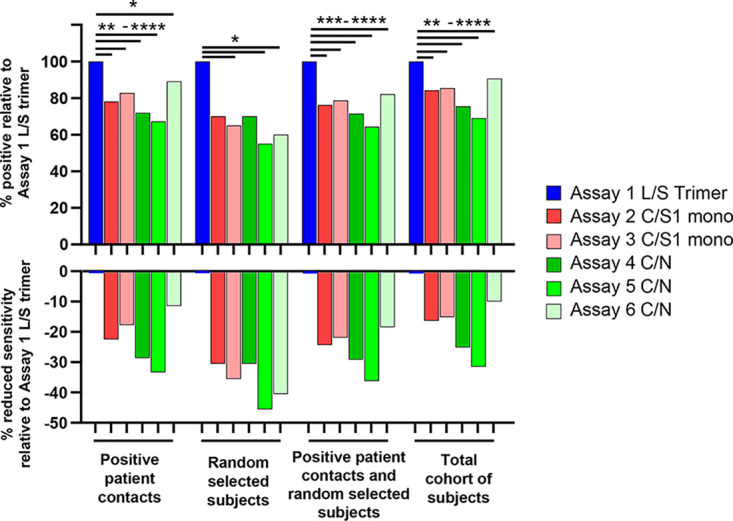
SARS-CoV-2-specific antibody responses to the trimeric S protein have significantly increased sensitivity compared to the S1 monomeric and/or N proteins in the postinfection population-based study. Analysis shows the percentage of seropositive subjects relative to the estimates obtained with the trimeric S protein (top) and the percentage of reduced sensitivity relative to the S1 monomeric and/or N proteins (bottom). Assays with blue bars used the S protein trimer as their bait for binding serum antibodies, while the red bars used the monomeric S1 protein and the green bars the N protein. Statistical analysis was performed using the McNemar test for matched participant samples: *, *P* < 0.045; **, *P* < 0.0022; ***, *P* < 0.0009; ****, *P* ≤ 0.0001.

With regard to the RT-PCR-positive group (*n* = 90 individuals) (Fig. 4, blue dots), the best sensitivity (96.7%) was found with the use of the trimeric S protein (assay 1 L/S trimer) compared to that of monomeric S and N proteins (Table 1). In the positive patient contact group (Fig. 4, red dots), the highest positivity rate (36.2%) was observed with the trimeric S protein, while positivity rates ranged between 32.2 and 24.3% with the other antigenic proteins (Fig. 4 and Table 1). With regard to the randomly selected group (Fig. 4, green dots; Table 1), we observed that anti-S antibody responses identified greater percentages of SARS-CoV-2-positive people (between 6.4 to 4.2%) than anti-N antibody responses (4.5 to 3.5%). Importantly, the pan-Ig assay using the N protein antigen (assay 6 C/N in Table 1) was the second most sensitive assay in the acute-infected cohort and in the RT-PCR-positive and positive patient contacts groups, but, conversely, was one of the least sensitive assays (3.9%) in detecting seropositive people randomly selected from the general population. The significantly higher sensitivity of the trimeric S protein antigen in the postinfection setting is highlighted in Fig. 5. Compared to the N and/or monomeric S antigens, the trimeric S protein antigen identified 10.9% to 32.8% more positive subjects in the positive patient contacts group, 30% to 45% more positive subjects in the randomly selected group, and 17.9% to 35.7% more positive subjects in a combined analysis of the positive patient contacts and randomly selected groups. Importantly, in terms of discordant detection of seropositive subjects in the postinfection cohort, sera that were confirmed in two or more commercial assays were also positives for assay 1 L/S trimer. In the overall postinfection cohort of 578 subjects, the trimeric S protein performed significantly better and detected between 9.4% and 31% more seropositive participants than the N and/or the S monomeric proteins (Fig. 5).

Taken together, these results indicate that anti-N antibody responses may substantially (i.e., 30% to 45%) underestimate the proportion of SARS-CoV-2 exposed individuals compared to anti-S antibody responses in population-based seroprevalence studies.

## DISCUSSION

Population-based seroprevalence studies are important to monitor the dynamics of the pandemic, to have a better appreciation of the number of infections, and to determine the proportion of the population that has developed specific SARS-CoV-2 immunity. Population-based seroprevalence studies performed in Switzerland, Spain, and New York City indicate that a minor percentage of the population, ranging from 10% to 20% of individuals, has been infected with SARS-CoV-2 (2–4, 19–21). The estimates of SARS-CoV-2-infected individuals from seroprevalence studies may be substantially influenced by qualitative and quantitative changes in the antibody responses during the transition from the acute to the postinfection phase, the clinical severity of the infection, and the antigenic protein used for the detection of the antibody responses.

SARS-CoV-2-specific antibodies (predominantly IgG) targeting either the S or the N proteins are generally assessed in both the acute and postinfection phases. The majority of studies and the validation of the assays with regard to the sensitivity and specificity has mostly been performed on cohorts from patients during the acute phase of infection (18, 19, 22, 23). The results from these studies indicate the use of N and S proteins were considered equally sensitive, with a generally higher sensitivity for the N protein in monitoring the development of antibody responses. Based on these observations in the acute phase of infection, it has been assumed that determination of antibody responses against the N or S proteins would be equally suitable in the postinfection phase for population-based seroprevalence studies. However, limited information is yet available on the evolution of the antibody response during the transition from the acute to the postinfection phase and, in particular, on the antibody responses against the two targets, the S and N proteins. Furthermore, the population-based studies comprise diverse populations of individuals, including RT-PCR-positive individuals with moderate to severe symptomatic infection who required hospitalization, RT-PCR-positive individuals with mild symptoms who did not require hospitalization, and pauci-/asymptomatic individuals with no previous RT-PCR confirmation of COVID-19 infection. Previous studies have shown that the magnitude of the antibody response may be influenced by the severity of the symptoms, with robust antibody response in patients with severe infection and weaker antibody response in patients with mild infection. Therefore, antibody responses can be lower in pauci-/asymptomatic individuals. For these reasons, population-based studies can be very challenging for estimating the proportion of SARS-CoV-2 infections in individuals who have experienced pauci-/asymptomatic infection.

Our results indicate a substantial drop in the sensitivity of antibody responses specific to the N protein, thus strongly suggesting a waning of these responses in the postinfection phase. In this regard, the estimated seroprevalence in the positive patient contacts and randomly selected groups is mostly impacted when only anti-N responses are assessed, with an underestimation ranging from 11% to 33% for the former and 30% to 45% for the latter group, compared to anti-S trimeric responses within the same groups.

Of note, the underestimation of SARS-CoV-2-seropositive individuals was also observed for antibody responses against monomeric S1 and was in the range of 18–22% in the positive patient contacts group and 30–35% in the randomly selected group samples. The greater sensitivity of antibody responses found against the trimeric S protein likely results from antibodies binding to the S2 subunit and the conservation of conformational epitopes within the higher order structure. This increased sensitivity was not obtained at the expense of cross-reactivity, since the specificity observed using the trimeric S protein was >99%. Overall, the underestimation of SARS-CoV-2-seropositive individuals was less important in the positive RT-PCR patients group, where it ranged from 1% to 26%.

The randomly selected subjects group provides valuable insights into the detection sensitivities of the assays using different SARS-CoV-2 antigens. However, one limitation in our study is that an insufficient number of donors were tested for a true population-based study. As such, the positive rate of 6.4% using the S trimer in the randomly selected subject group is insufficiently powered to estimate population seroprevalence.

A recent study (24) has shown that 40% of asymptomatic individuals became seronegative over time. However, anti-N antibody responses were determined in this study. Based on our results, it is likely that the loss of antibody response observed is due to the selective waning of the anti-N antibody responses rather than to a global reduction of the SARS-CoV-2 antibody response. Furthermore, the present findings are also important for the development of appropriate monitoring strategies for the evaluation and development of vaccines against SARS-CoV-2.

In conclusion, these results provide new insights into the evolution of the SARS-CoV-2 antibody response from the acute to the postinfection phase and indicate that the detection of antibody responses against the native trimeric S protein should be implemented to avoid large underestimation of SARS-CoV-2 infections in population-based seroprevalence studies.

## MATERIALS AND METHODS

### Study populations.

**(i) Patients with acute infections.** Comparison of assays for the acute/subacute phase of infection was performed on 161 sera, including the following: (i) 93 sera expected to be positive, sampled from hospitalized patients with severe to moderate symptoms at 0 to 45 days post-onset of the symptoms and documented with a positive SARS-CoV-2 RT-PCR test; (ii) 65 sera expected to be negative, sampled before November 2019, presented as pre-COVID-19 sera, and including 18 samples from patients documented positive for a human coronavirus (E229, OC43, HKU1, or NL63) RT-PCR. Dates of the onset of symptoms were extracted from the electronic record of the 93 SARS-CoV-2 RT-PCR-positive patients.

**(ii) Postinfection cohort.** A second comparison of assays was performed on sera from the seroprevalence study of the Vaud Canton in Switzerland (SerocoViD) performed by the Centre for Primary Care and Public Health, University of Lausanne (Unisanté). Out of the 1,942 participants who provided a blood sample between 4 May and 27 June 2020, a subset of 578 subjects was included in the present analysis, categorized as follows: (i) 90 subjects were expected to be positive, i.e., sampled from mostly mild to paucisymptomatic patients (only 21% had been hospitalized) documented with a positive SARS-CoV-2 RT-PCR test; (ii) 177 were sampled from contacts of RT-PCR-positive subjects; and (iii) 311 were randomly selected subjects in the general population. In the latter group, the addresses of subjects recruited were randomly selected by the Swiss Federal Office of Statistics from the population registry of the canton of Vaud in Switzerland. There were 304 women (52.6%) with a mean age of 39.2 years (SD 24.2, range: 6 months to 90 years) and 271 men (46.9%) with a mean age of 39.9 years (SD 24.7, range: 13 months to 90 years). Three donors were not defined by gender.

**(iii) Pre-COVID-19 pandemic donors.** Negative-control serum samples from 256 adult healthy donors with ages ranging from 18 to 81 years of age were collected prior to November 2019 as part of the Swiss Immune Setpoint study sponsored by the Swiss Vaccine Research Institute. Specificity tests for the Luminex S-protein assay with a diverse set of 108 patient sera included the 65 sera collected prior to November 2019 and used in the blinded tests performed with all six assays and an additional 43 prepandemic patient samples. This diverse set of samples consisted of sera from pregnant women (*n* = 14), prepandemic coronavirus-infected donors (OC43, E229, NL63, and HKU1; *n* = 19), patients with infectious diseases (HIV, rubella, HSV1, HSV2, RSV, CMV, EBV, influenza, and varicella; *n* = 57), and patients with autoimmune diseases, including lupus (*n* = 18). Study design and use of subject sera samples were approved by the Institutional Review Board of the Lausanne University Hospital and the Commission d’éthique du Canton de Vaud (CER-VD) stated that authorization was not required.

### Preparation of Luminex beads.

Luminex beads used for the serological binding assays were prepared by covalent coupling of SARS-CoV-2 proteins with MagPlex beads using the manufacture’s protocol with a Bio-Plex Amine Coupling kit (Bio-Rad, France). Briefly, 1 ml of MagPlex-C microspheres (Luminex) were washed with wash buffer and then resuspended in activation buffer containing a freshly prepared solution of 1-ethyl-3-(3-dimethylaminopropyl) carbodiimide (EDC) and *N*-hydroxysulfosuccinimide (S-NHS), (Thermo Fisher Scientific, USA). Activated beads were washed in phosphate-buffered saline (PBS) followed by the addition of 50 μg of protein antigen. The coupling reaction was performed at 4°C overnight with bead agitation using a Hula-Mixer (Thermo Fisher). Beads were then washed with PBS, resuspended in blocking buffer, and then incubated for 30 min with agitation at room temperature. Following a final PBS washing step, beads were resuspended in 1.5 ml of storage buffer and kept protected from light in an opaque tube at 4°C. Each of the SARS-CoV-2 proteins was coupled with different colored MagPlex beads so that tests could be performed with a single protein bead per well or in a multiplexed Luminex serological binding assay.

### SARS-CoV-2 proteins evaluated in Luminex assay.

The S protein trimer was designed to mimic the native trimeric conformation of the protein *in vivo* and the expression vector was kindly provided by Jason McLellan, University of Texas, Austin, Texas. The vector encoded the prefusion ectodomain of the SARS-CoV-2 spike protein with a C-terminal T4 foldon fusion domain to stabilize the trimer complex, along with C-terminal 8×-His and 2×-Strep tags for affinity purification. The trimeric spike protein was transiently expressed in suspension-adapted ExpiCHO cells (Thermo Fisher) in ProCHO5 medium (Lonza) at 5 × 10^6^ cells/ml using PEI MAX (Polysciences) for DNA delivery. At 1 h posttransfection, dimethyl sulfoxide (DMSO; AppliChem) was added to 2% (vol/vol). Following a 7-day incubation with agitation at 31°C and 4.5% CO2, the cell culture medium was harvested and clarified using a 0.22-μm filter. The conditioned medium was loaded onto Streptactin (IBA) and StrepTrap HP (Cytiva) columns in tandem, washed with PBS, and eluted with 10 mM desthiobiotin in PBS. The purity of the S protein trimer was determined to be >99% pure by SDS-PAGE analysis.

Receptor-binding domain (RBD) and S1 SARS-CoV-2 proteins were prepared as previously described (5). Upon initial characterization of the assays, serum dilutions of 1:50 down to 1:2,700 were evaluated for SARS-CoV-2 PCR-positive subjects and healthy donors. A 1:300 dilution of serum was selected for screening patient samples, as it gave a high mean fluorescence intensity (MFI) signal for all donors and a low background staining with serum samples from pre-COVID-19 pandemic healthy donors. In addition to the high positive signal and low background, <1 μl of serum was needed to evaluate anti-SARS-CoV-2 antibody binding in the Luminex assay binding assays.

### Luminex anti-SARS-CoV-2 antibody binding assay.

Luminex beads coupled with the spike, RBD, or S1 proteins were diluted 1:100 in PBS with 50 μl added to each well of a Bio-Plex Pro 96-well flat bottom plates (Bio-Rad). Following bead washing with PBS on a magnetic plate washer (MAG2x program), 50 μl of individual serum samples diluted at 1:300 in PBS was added to the plate wells. Along with samples, three replicates of a 1:300 negative-control pool of pre-COVID-19 pandemic healthy human sera (BioWest human serum AB males; VWR) were evaluated on each 96-well plate. Plates were sealed with adhesive film, protected from light with a dark cover and agitated at 500 rpm for 60 min on a plate shaker. Beads were then washed on the magnetic plate washer and anti-human IgG-PE (phycoerythrin) secondary antibody (OneLambda Thermo Fisher) was added at a 1:100 dilution with 50 μl per well. Plates were agitated for 45 min and then washed on the magnetic plate washer. Beads resuspended in 80 μl of reading buffer were agitated 5 min at 700 rpm on the plate shaker then read directly on a Luminex FLEXMAP 3D plate reader (Thermo Fisher). The MFI signal for each test serum sample was divided by the mean signal for the negative-control samples to yield an MFI ratio that normalized values between plates and between different Luminex instruments tested. Considering that two of the three false positives from the 364 SARS-CoV-2 negative donors had MFI signals of less than 6 (Fig. 1A), an additional criterion for positivity was established for large general population screens, including the postinfection cohort. Here, sera with signal intensities between 4 and 6 were defined as being at the limit of positivity, which increases the assay sensitivity to 99.7% with only one acute HIV-infected subject having a 6.8 MFI signal.

### Immunoassays.

The new Luminex S protein trimer IgG assay (assay 1 L/S trimer) was compared with five commercially available SARS-CoV-2 immunoassays: (i) two ELISAs from EuroImmun (assay 3 C/S1 mono) and Epitope Diagnostics (assay 4 C/N) detecting IgG against the S1 and N proteins, respectively; (ii) two CLIAs from Diasorin (assay 2 C/S1 mono) and Snibe (assay 5 C/N) detecting IgG against S1 protein and N protein + S antigen peptide, respectively; and (iii) a pan-Ig ECLIA from Roche (assay 6 C/N) targeting the N protein. The Snibe assay was grouped with the N protein assays in our analysis since it contained only a portion of the S1 protein.

ELISA and CLIA were performed according to the manufacturers’ instructions. EuroImmun and Epitope Diagnostic IgG ELISA were done manually as per protocol, with the exception of the washing steps performed with a microplate washer (PW40, Bio-Rad, France). Optical densities (OD) were measured with a microplate reader (800 TSI, BioTek, USA). Each sample was measured in duplicate. The LIAISON SARS-CoV-2 IgG kit was performed on a Liaison XL (Diasorin, Italy), and the MAGLUMI 2019-nCoV IgG and IgM kits were performed on a MAGLUMI 800 (Snibe, China). The Elecsys anti-SARS-CoV-2 assay was performed on a COBAS 6000 (Roche, Switzerland).

### Statistical analyses.

The sensitivities of the different assays were calculated according to the day post-symptoms on expected positive sera taken from patients with a positive RT-PCR. The RT-PCR was previously performed according to Corman et al (25) on our automated molecular diagnostic platform (26).

Sensitivity and specificity of the assays with 95% confidence interval (CI) (Wilson/Brown method of GraphPad Prism 8.3.0) were calculated with MS Excel and GraphPad prism. For comparisons between the Luminex assay and the five other serological assays, *R*^2^ values were calculated using the Pearson test and the McNemar test was used to determine the *P* value of significant differences for sensitivities in detecting seropositive subjects in the patient subsets within the postinfection cohort. All statistical analyses were done with GraphPad prism.

## Supplementary Material

Supplemental file 1

## References

[1] World Health Organization. 2020. Coronavirus disease (COVID-2019) situation reports-175. World Health Organization, Geneva, Switzerland.

[2] Norheim OF. 2020. Protecting the population with immune individuals. Nat Med 26:823–824. doi:10.1038/s41591-020-0896-2. 32382153

[3] Phelan AL. 2020. COVID-19 immunity passports and vaccination certificates: scientific, equitable, and legal challenges. Lancet 395:1595–1598. doi:10.1016/S0140-6736(20)31034-5. 32380041 PMC7198144

[4] Weitz JS, Beckett SJ, Coenen AR, Demory D, Dominguez-Mirazo M, Dushoff J, Leung C-Y, Li G, Măgălie A, Park SW, Rodriguez-Gonzalez R, Shivam S, Zhao CY. 2020. Modeling shield immunity to reduce COVID-19 epidemic spread. Nat Med 26:849–854. doi:10.1038/s41591-020-0895-3. 32382154 PMC8272982

[5] Okba NMA, Muller MA, Li W, Wang C, GeurtsvanKessel CH, Corman VM, Lamers MM, Sikkema RS, de Bruin E, Chandler FD, Yazdanpanah Y, Le Hingrat Q, Descamps D, Houhou-Fidouh N, Reusken C, Bosch BJ, Drosten C, Koopmans MPG, Haagmans BL. 2020. Severe acute respiratory syndrome coronavirus 2-specific antibody responses in coronavirus disease patients. Emerg Infect Dis 26:1478–1488. doi:10.3201/eid2607.200841. 32267220 PMC7323511

[6] Li G, Chen X, Xu A. 2003. Profile of specific antibodies to the SARS-associated coronavirus. N Engl J Med 349:508–509. doi:10.1056/NEJM200307313490520. 12890855

[7] Zhao J, Yuan Q, Wang H, Liu W, Liao X, Su Y, Wang X, Yuan J, Li T, Li J, Qian S, Hong C, Wang F, Liu Y, Wang Z, He Q, Li Z, He B, Zhang T, Fu Y, Ge S, Liu L, Zhang J, Xia N, Zhang Z. 2020. Antibody responses to SARS-CoV-2 in patients of novel coronavirus disease 2019. Clin Infect Dis doi:10.1093/cid/ciaa344. PMC7184337 32221519

[8] Meyer B, Drosten C, Muller MA. 2014. Serological assays for emerging coronaviruses: challenges and pitfalls. Virus Res 194:175–183. doi:10.1016/j.virusres.2014.03.018. 24670324 PMC7114385

[9] Infantino M, Damiani A, Gobbi FL, Grossi V, Lari B, Macchia D, Casprini P, Veneziani F, Villalta D, Bizzaro N, Cappelletti P, Fabris M, Quartuccio L, Benucci M, Manfredi M. 2020. Serological assays for SARS-CoV-2 infectious disease: benefits, limitations and perspectives. Isr Med Assoc J 22:203–210. 32286019

[10] Chia WN, Tan CW, Foo R, Kang AEZ, Peng Y, Sivalingam V, Tiu C, Ong XM, Zhu F, Young BE, Chen MI, Tan YJ, Lye DC, Anderson DE, Wang LF. 2020. Serological differentiation between COVID-19 and SARS infections. Emerg Microbes Infect 9:1497–1505. doi:10.1080/22221751.2020.1780951. 32529906 PMC7473126

[11] Cao WC, Liu W, Zhang PH, Zhang F, Richardus JH. 2007. Disappearance of antibodies to SARS-associated coronavirus after recovery. N Engl J Med 357:1162–1163. doi:10.1056/NEJMc070348. 17855683

[12] Choe PG, Perera R, Park WB, Song KH, Bang JH, Kim ES, Kim HB, Ko LWR, Park SW, Kim NJ, Lau EHY, Poon LLM, Peiris M, Oh MD. 2017. MERS-CoV antibody responses 1 year after symptom onset, South Korea, 2015. Emerg Infect Dis 23:1079–1084. doi:10.3201/eid2307.170310. PMC5512479 28585916

[13] Wu LP, Wang NC, Chang YH, Tian XY, Na DY, Zhang LY, Zheng L, Lan T, Wang LF, Liang GD. 2007. Duration of antibody responses after severe acute respiratory syndrome. Emerg Infect Dis 13:1562–1564. doi:10.3201/eid1310.070576. 18258008 PMC2851497

[14] Payne DC, Iblan I, Rha B, Alqasrawi S, Haddadin A, Al Nsour M, Alsanouri T, Ali SS, Harcourt J, Miao C, Tamin A, Gerber SI, Haynes LM, Al Abdallat MM. 2016. Persistence of antibodies against Middle East Respiratory Syndrome Coronavirus. Emerg Infect Dis 22:1824–1826. doi:10.3201/eid2210.160706. 27332149 PMC5038413

[15] Guo X, Guo Z, Duan C, Chen Z, Wang G, Lu Y, Li M, Lu J. 2020. Long-term persistence of IgG antibodies in SARS-CoV infected healthcare workers. medRxiv doi:10.1101/2020.02.12.20021386.

[16] Wrapp D, Wang N, Corbett KS, Goldsmith JA, Hsieh CL, Abiona O, Graham BS, McLellan JS. 2020. Cryo-EM structure of the 2019-nCoV spike in the prefusion conformation. bioRxiv doi:10.1101/2020.02.11.944462. PMC7164637 32075877

[17] Coste AT, Jaton K, Papadimitriou-Olivgeris M, Greub G, Croxatto A. 2020. Comparison of SARS-CoV-1 2 serological tests with different antigen targets. medRxiv doi:10.1101/2020.07.09.20149864. PMC7670982 33253926

[18] Kohmer N, Westhaus S, Ruhl C, Ciesek S, Rabenau HF. 2020. Brief clinical evaluation of six high-throughput SARS-CoV-2 IgG antibody assays. J Clin Virol 129:104480. doi:10.1016/j.jcv.2020.104480. 32505777 PMC7263247

[19] Stringhini S, Wisniak A, Piumatti G, Azman AS, Lauer SA, Baysson H, De Ridder D, Petrovic D, Schrempft S, Marcus K, Yerly S, Arm Vernez I, Keiser O, Hurst S, Posfay-Barbe KM, Trono D, Pittet D, Getaz L, Chappuis F, Eckerle I, Vuilleumier N, Meyer B, Flahault A, Kaiser L, Guessous I. 2020. Seroprevalence of anti-SARS-CoV-2 IgG antibodies in Geneva, Switzerland (SEROCoV-POP): a population-based study. Lancet 396:313–319. doi:10.1016/S0140-6736(20)31304-0. 32534626 PMC7289564

[20] Saplakoglu Y. 2020. 1 in 5 people tested in New York City had antibodies for the coronavirus. LiveScience.

[21] Anonymous. Gobierno de España, Ministerio de Sanidad. Estudio ENE-COVID19: primera ronda. Estudio nacional de sero-epidemiología de la infección por SARS-CoV-2 en España.

[22] Coste AT, Jaton K, Papadimitriou-Olivgeris M, Croxatto A, Greub G. 2020. Indication for SARS-CoV-2 serology: first month follow-up. medRxiv doi:10.1101/2020.06.30.20140715.

[23] Montesinos I, Gruson D, Kabamba B, Dahma H, Van den Wijngaert S, Reza S, Carbone V, Vandenberg O, Gulbis B, Wolff F, Rodriguez-Villalobos H. 2020. Evaluation of two automated and three rapid lateral flow immunoassays for the detection of anti-SARS-CoV-2 antibodies. J Clin Virol 128:104413. doi:10.1016/j.jcv.2020.104413. 32403010 PMC7198434

[24] Long QX, Tang XJ, Shi QL, Li Q, Deng HJ, Yuan J, Hu JL, Xu W, Zhang Y, Lv FJ, Su K, Zhang F, Gong J, Wu B, Liu XM, Li JJ, Qiu JF, Chen J, Huang AL. 2020. Clinical and immunological assessment of asymptomatic SARS-CoV-2 infections. Nat Med 26:1200–1204. doi:10.1038/s41591-020-0965-6. 32555424

[25] Corman VM, Landt O, Kaiser M, Molenkamp R, Meijer A, Chu DK, Bleicker T, Brunink S, Schneider J, Schmidt ML, Mulders DG, Haagmans BL, van der Veer B, van den Brink S, Wijsman L, Goderski G, Romette JL, Ellis J, Zambon M, Peiris M, Goossens H, Reusken C, Koopmans MP, Drosten C. 2020. Detection of 2019 novel coronavirus (2019-nCoV) by real-time RT-PCR. Euro Surveill 25:2000045. doi:10.2807/1560-7917.ES.2020.25.3.2000045. PMC6988269 31992387

[26] Greub G, Sahli R, Brouillet R, Jaton K. 2016. Ten years of R&D and full automation in molecular diagnosis. Future Microbiol 11:403–425. doi:10.2217/fmb.15.152. 27028061

