# Supplementary material for: Changes in SARS-CoV-2 Spike versus Nucleoprotein Antibody Responses Impact the Estimates of Infections in Population-Based Seroprevalence Studies
Source: J Virol. 2021 Jan 13;95(3):e01828-20. doi: 10.1128/JVI.01828-20 (PMC7925109; doi:10.1128/JVI.01828-20)
Supplement: Supplemental file 1 [file JVI.01828-20-s0001.pdf]

Supplemental Figures and Table

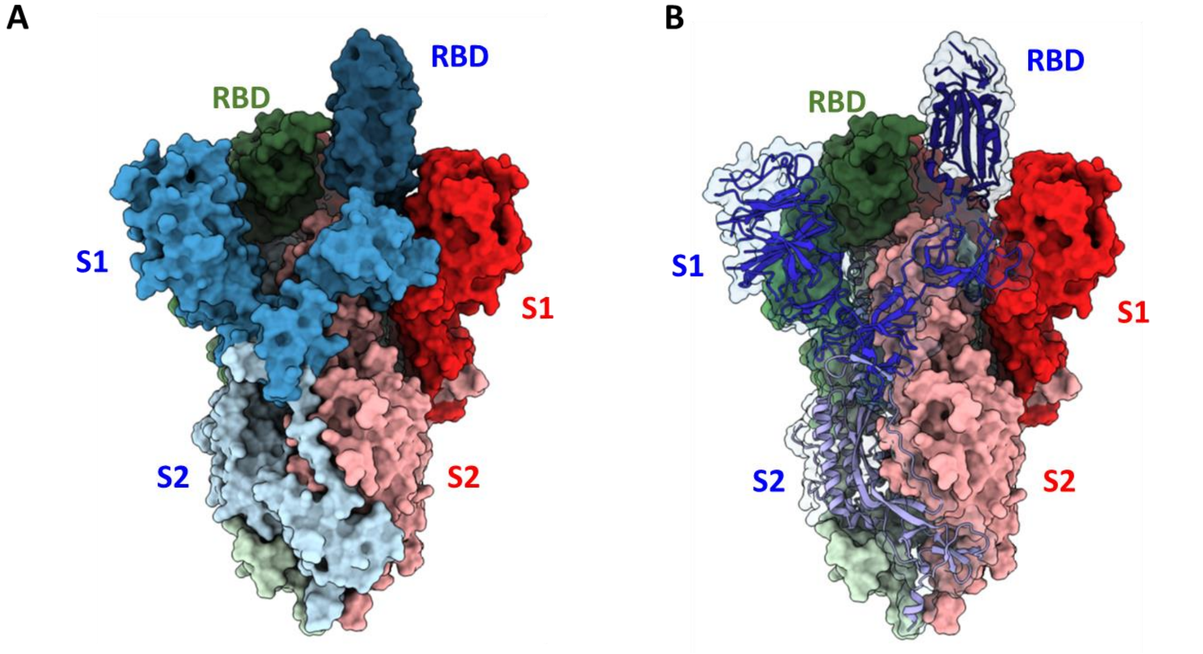

Supplementary Figure 1: Structural representation of the SARS-CoV-2 S protein Trimer. A) Space filled representation of the S protein with trimer subunits shown in blue, red and green (PDB 6VSB). The labeled S2, S1 and RBD portions of each subunit are in light, mid and dark colors, respectively. Compared to the monomeric S1 protein, this image demonstrates that the native S protein trimer, consisting of S1 and S1 proteins, that has significantly greater conformational epitopes for antibody binding that are only present in the higher order structure. B) S protein trimer with the blue subunit represented as a ribbon structural.

A

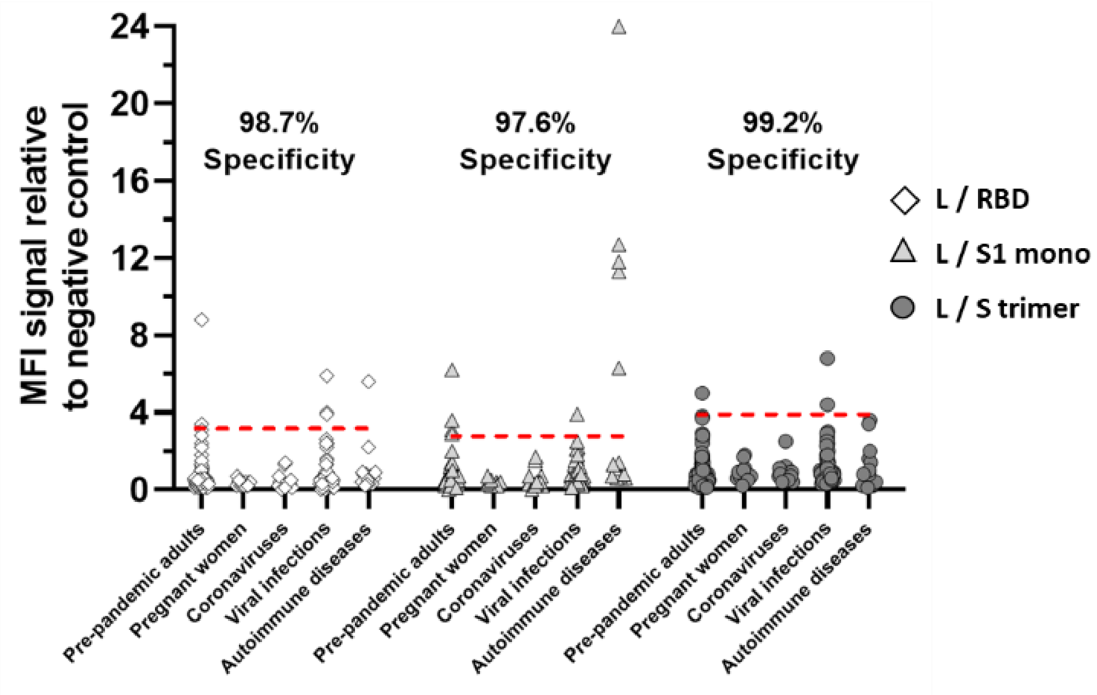
MFI signal relative to negative control
24
20
16
12
8
4
0
98.7% Specificity
97.6% Specificity
99.2% Specificity
L / RBD
L / S1 mono
L / S trimer
Pre-pandemic adults
Pregnant women
Coronaviruses
Viral infections
Autoimmune diseases

B

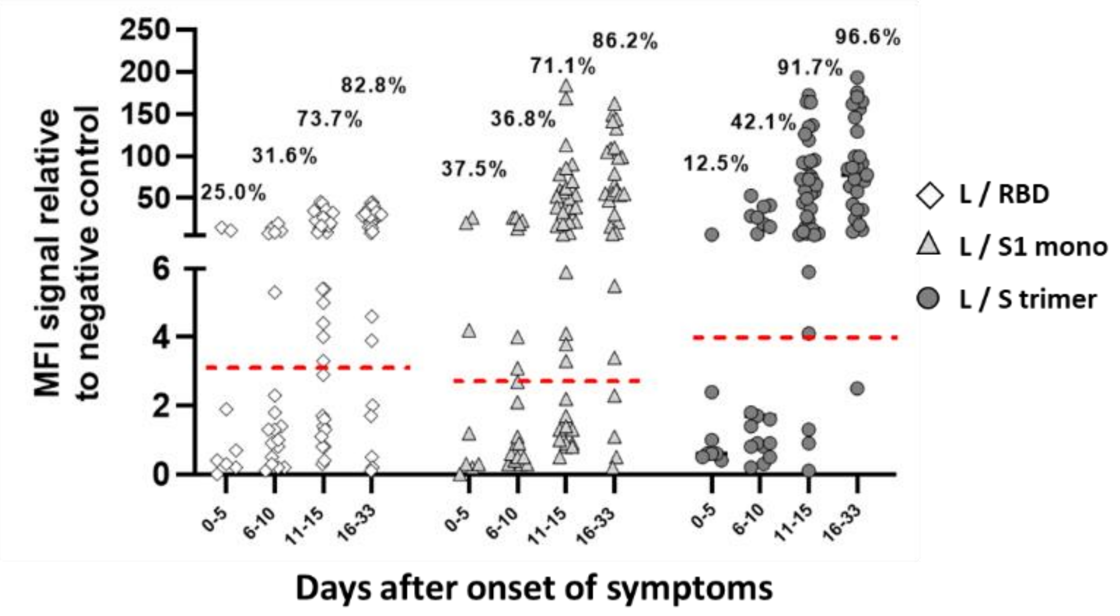
MFI signal relative to negative control
250
200
150
100
50
6
4
2
0
25.0%
31.6%
73.7%
82.8%
37.5%
36.8%
71.1%
86.2%
12.5%
42.1%
91.7%
96.6%
L / RBD
L / S1 mono
L / S trimer
0-5
6-10
11-15
16-33
Days after onset of symptoms

Supplementary Figure 2: SARS-CoV-2-specific IgG binding antibody responses against the RBD domain, monomeric S1 protein and trimeric S protein in a Luminex binding assay. A) Specificity was assessed using the cohort of negative control sera described in Figure 1A (Pre-pandemic adults, n=256; pregnant women, n=14; pre-pandemic coronaviruses, n=19; viral infection patients, n=57; autoimmune diseases, n=18). B) Sensitivity was determined with the 92 acute infected serum samples described in Figure 1B (Days 0 to 5, n=8; Days 6-10, n=19; Days 11-15, n=36; Days 15-33, n=29). The cut-off for positivity used in the RBD, S1 monomer and S protein trimer Luminex IgG assays are 3.2-, 2.8- and 4.0-fold over the negative control, respectively and were established by using mean value + 4×SD of each for the 356 pre-COVID-19 pandemic serum samples in A.

Supplementary Table 1. Cumulative data of SARS-CoV-2-specific IgG antibody responses on sera collected during the acute infection from hospitalized patients with moderate to severe symptoms

Test	Days post-onset of symptoms	N° sera from patients RT-PCR positive		IgG** Se	IgG** Se 95% CI	N° sera pre-COVID*		IgG** Sp	IgG** Sp 95% CI
		Total	Positive in serology			Total	Positive in serology		
**Test 1 L/S Trimer**	All	92	70	0.76	0.66 to 0.83	65	0	1	0.94 to 1.0
	0-5	8	1	0.13	0.0064 to 0.47				
	6-10	19	8	0.42	0.23 to 0.64				
	11-15	36	33	0.92	0.78 to 0.97				
	16-33	29	28	0.97	0.83 to 1.0				
**Test 2 C/S1 mono**	All	93	49	0.53	0.43 to 0.63	65	0	1	0.95 to 1.0
	0-5	8	0	0	0.0 to 0.32				
	6-10	19	4	0.21	0.085 to 0.43				
	11-15	37	21	0.57	0.41 to 0.71				
	16-33	29	24	0.83	0.65 to 0.92				
**Test 3 C/S1 mono**	All	89	58	0.65	0.55 to 0.74	65	2	0.97	0.90 to 0.99
	0-5	8	1	0.13	0.0064 to 0.47				
	6-10	18	5	0.28	0.12 to 0.51				
	11-15	34	27	0.79	0.63 to 0.90				
	16-33	29	25	0.86	0.69 to 0.95				
**Test 4 C/N**	All	90	67	0.74	0.64 to 0.82	65	1	0.98	0.92 to 1.0
	0-5	7	1	0.14	0.0073 to 0.51				
	6-10	18	8	0.44	0.25 to 0.66				
	11-15	36	32	0.89	0.75 to 0.96				
	16-33	29	26	0.89	0.74 to 0.96				
**Test 5 C/N**	All	93	59	0.63	0.53 to 0.73	65	0	1	0.95 to 1.0
	0-5	8	0	0	0.0 to 0.32				
	6-10	19	6	0.32	0.15 to 0.54				
	11-15	37	27	0.73	0.57 to 0.85				
	16-33	29	26	0.9	0.74 to 0.96				
**Test 6 C/N**	All	93	68	0.73	0.63 to 0.81	65	0	1	0.95 to 1.0
	0-5	8	0	0	0.0 to 0.32				
	6-10	19	9	0.47	0.27 to 0.68				
	11-15	37	32	0.86	0.72 to 0.94				
	16-33	29	27	0.93	0.78 to 0.99				

Se: sensitivity; Sp: specificity ; * serum sampled before 1st November 2019 considered as "negative"; **IgG, except for the Test 6 N, which corresponds to a pan-Ig assay
